# Ovarian cancer immunotherapy: opportunities, progresses and challenges

**DOI:** 10.1186/1756-8722-3-7

**Published:** 2010-02-10

**Authors:** Bei Liu, John Nash, Carolyn Runowicz, Helen Swede, Richard Stevens, Zihai Li

**Affiliations:** 1 Department of Immunology, University of Connecticut School of Medicine, Farmington, USA; 2 Neag Comprehensive Cancer Center, University of Connecticut School of Medicine, Farmington, USA; 3 Department of Community Medicine & Health Care, University of Connecticut School of Medicine, Farmington, USA

## Abstract

Due to the low survival rates from invasive ovarian cancer, new effective treatment modalities are urgently needed. Compelling evidence indicates that the immune response against ovarian cancer may play an important role in controlling this disease. We herein summarize multiple immune-based strategies that have been proposed and tested for potential therapeutic benefit against advanced stage ovarian cancer. We will examine the evidence for the premise that an effective therapeutic vaccine against ovarian cancer is useful not only for inducing remission of the disease but also for preventing disease relapse. We will also highlight the questions and challenges in the development of ovarian cancer vaccines, and critically discuss the limitations of some of the existing immunotherapeutic strategies. Finally, we will summarize our own experience on the use of patient-specific tumor-derived heat shock protein-peptide complex for the treatment of advanced ovarian cancer.

## Introduction

Ovarian cancer occurs with a lifetime incidence in approximately 1 in 58 women and it is the fifth leading cause of cancer death in women and is the leading cause of death among gynecologic cancers. It is estimated that approximately 21,550 new cases of ovarian cancer were diagnosed in 2009 in the United States with 14,600 deaths[1]. Sixty-seven percent of patients are diagnosed at stages III and IV, with resultant low relative-survival rates[1] despite, in many cases, apparently optimal surgery followed by the most effective combination chemotherapies available to date. Therefore, there is a compelling need for innovative and effective therapies.

Malignant tumors have been shown to be immunogenic in some cancer sites, including ovarian cancer. Some of the strongest evidence linking anti-tumor immunity and cancer have been made in ovarian cancer [2-5]. Understanding how the immune response is activated in ovarian cancer is a prerequisite for designing clinically meaningful immunologic strategies against this disease. Over the last two decades, there have been numerous clinical trials in ovarian cancer using immunologic modalities[6]. Results have been at best mixed, which demonstrates the need for a thoughtful and integrative approach to examine the role of immunotherapy in this disease. In this article, we will examine several key issues in this rapidly evolving area, highlighting the opportunities and challenges. We hope that our work will provide an overview and contribute to discovery the most effective immunotherapy of ovarian cancer.

## Historical Perspective: Is Cancer Immunogenic?

Immunogenicity is the ability of antigens to elicit an immune response. It is well known that traditional vaccines can be very powerful in the prevention of infectious diseases such as smallpox. The early vaccines against smallpox, originating in China, were inspired by the concept of variolation. The term *vaccine *(adopted from the Latin *vaccin-us*, from *vacca cow*) derives from Edward Jenner's use of cow pox particulate, which was found to provide protection against smallpox when it was administered to humans around 1796. Nearly 100 years ago, Paul Ehrlich proposed his theory of "immune surveillance", where tumor cells are rapidly eliminated by the immune system on a daily basis. This concept could not be tested at that time due to lack of appropriate models and *in vitro *systems. Even immunodeficient mouse models have failed to provide direct and definitive evidence supporting this theory[7].

The first cancer vaccine in human is attributed to William Coley in 1893[8]. He observed that some patients with cancer benefited from bacterial infection resulting in tumor shrinkage. This prompted him to treat the patients with bacterial extracts. This novel observation led many to conclude that the immune system can recognize tumor-associated antigens. Indirect or circumstantial evidences are now mounting supporting the existence of the cancer immunosurveillance mechanism in both animals and humans. However, cancer also adopts a variety of strategies to evade or suppress the immune system. The host-cancer interaction may or may not lead to tumor eradication. Thus the concept of "cancer immunosurveillance" is being replaced by the concept of "cancer immunoediting," which emphasizes a dynamic process of interaction between cancer and the immune system. Operationally, cancer immunoediting can be divided arbiturilly into three phases: elimination, equilibrium, and escape, highlighting the dynamic interaction between the host immune system and cancer. In the early phase of tumor initiation, immune response is effective, resulting in elimination of cancer. This is followed by a long period of equilibrium when cancer is not eliminated but it is kept in check by the immune system and is thus not clinically detectable. Cancer becomes clinically detectable when it has escaped effective anti-tumor immunity. This concept would predict that the immune system not only protects the host against the development of primary cancer, but also sculpts tumor immunogenicities, a process which has been experimentally confirmed[7].

Initially tumor antigens were broadly classified into two categories based on their pattern of expression: tumor-specific antigens (TSA), which are present only on tumor cells and not on any other cells; and tumor-associated antigens (TAA), which are present on some tumor cells and also some normal cells. However, this classification is imperfect because many antigens that were thought to be tumor-specific turned out to be expressed on some normal cells as well. The modern classification of tumor antigens is based on their molecular structure and source. Several techniques to identify tumor antigens have been developed, which include serological identification of antigens by recombinant cDNA expression cloning (SEREX)[9,10], T-cell epitope cloning (TEPIC), and bioinformatics[11]. A large array of immunogenic tumor antigens has been identified. Currently, human tumor antigens are classified into the following classes: differentiation antigens, overexpression/amplification antigens, mutational antigens, cancer testis antigens, oncofetal antigens, and viral antigens[6] (Table 1). Up to now, over 1,000 human tumor antigens have been established in a human cancer immunome database http://ludwig-sun5.unil.ch/CancerImmunomeDB/. This effort aims to enhance the opportunity for researchers in the cancer immunology field to design efficacious immunotherapy strategies through specificically targeted tumor antigens.

**Table 1:** Human Tumor-Associated Antigens*

Antigen category	Antigens	Tumor type	Vaccine	Reference
Differentiation Antigens	Tyrosinase	Melanoma	Yes	Int J Cancer 1996;67:54[60]
	Melan- Mart-1	Melanoma	Yes	Cancer J Sci Am 1997; 3:37[61]
	gp-100	Melanoma	Yes	Nat Med 1998; 4:321[62]
				
Overexpression/Amplification	HER-2/neu	Ovarian cancer Breast cancer	Yes	J Clin Oncol. 2002; 20:2624[13]
Antigens	p53	various tumors	Yes	J Immunol 1998; 160:328[63] Cancer Immunol Immunother. 2004; 53:633[64]
				
Mutational Antigens	p53	various tumors	Yes	J Clin Oncol 2005; 23:5099[65]
	Ras	various tumors	Yes	Int J Cancer 2001; 92:441[66]
				
Cancer Testis Antigens	MAGE	Melanoma	Yes	Int J Cancer 1999; 80:219[67]
	NY-ESO-1	Ovarian cancer	Yes	Clin Cancer Res. 2008; 14:2740[31]
	LAGE-1	Ovarian cancer Melanoma Bladder cancer	No	Cancer Res. 2003; 63:6076[20]
				
Glycolipid Antigens	MUC-1	Adenocarcinoma	Yes	J. Clin. Invest. 1997; 100:2783[68]
	MUC-16 (CA125)	Ovarian cancer	Yes	Int J Cancer 2002; 98:737[69] Clin Cancer Res. 2004; 22:3507[70]
				
Oncofetal Antigens	AFP	Germ cell tumors	No	Gynecol. Oncol. 2000; 77:203[71]
	CEA	Colorectal cancer	Yes	Ann Surg Oncol 1996; 3:495[72]
	PSA	Prostate cancer	Yes	Urology 1999; 53:260[73]
				
Viral Antigens	HPV	Cervical cancer	Yes	Lancet 1996; 347:1523[74]

*This represents only a partial list of tumor antigens for immunotherapy.

## Clinical Evidence for the Role of Immunosurveillance Against Human Ovarian Cancer

### Intratumoral T cells correlate with clinical outcome

The first evidence of the role of immunosurveillance against human ovarian cancer was the presence of tumor-infiltrating lymphocytes (TILs), which correlated positively and strongly with patient survival[2]. Zhang *et al*. (2003) performed immunohistochemical analyses to assess the distribution of TILs in 186 frozen specimens from stage III or IV ovarian cancers and conducted clinical outcome analyses. In this study, CD3^+ ^TILs were detected within tumor-cell islets in 102 of the 186 tumors (54.8%), whereas CD3^+ ^TILs were not detected in 72 of 186 tumors (38.7%); 12 tumors could not be evaluated (6.5%). They also assessed the number of CD4^+ ^and CD8^+ ^T cells in 30 tumors, and the numbers of CD4^+ ^and CD8^+ ^T cells were closely correlated (R^2 ^= 0.66, p < 0.001). The immunohistochemical staining data showed that intratumoral CD4^+ ^and CD8^+ ^cells were either both present or both absent. Patients whose tumors contained TILs had five-year overall survival rates of 38%, whereas patients whose tumors lacked TILs only had five-year overall survival rates of 4.5%. The five-year progression-free survival rates for patients whose tumors were present and absent of TILs were 31.0% and 8.7% respectively. Thus, overall and progression-free five-year survival rates were significantly prolonged in the patients whose tumors contained TILs compared to the patients whose tumors did not contain TILs (p < 0.001 for both comparisons). In a multivariate analysis, it was shown that the presence or absence of TILs (*p *< 0.001) and the extent of residual tumor (*p *< 0.001) correlated with overall and progression-free survival, but patient age (<55 years vs. >55 years), tumor grade (grade 1 vs. grade 3, grade 2 vs. grade 3), and type of first-line chemotherapy did not vary with outcomes [2].

Other studies have confirmed that the intraepithelial CD3^+ ^TIL count is a significant prognostic factor in epithelial ovarian cancer (EOC). Tomšová *et al*. showed improved overall survival among 116 EOC patients with higher versus lower counts of intraepithelial CD3^+ ^TILs (> 60 vs. 29 months, respectively, *p *< 0.0001)[3].

### Predictable value of tumor infiltrating regulatory T cells

Sato *et al*. performed immunohistochemical analyses for TILs in 117 cases of epithelial ovarian cancer. Patients with higher frequencies of intraepithelial CD8^+ ^T cells demonstrated improved survival compared to patients with lower frequencies (55 vs. 26 months; hazard ratio = 0.33; 95% C.I., 0.18-0.60; *p *= 0.0003). In addition, the subgroups with high versus low intraepithelial CD8^+^/CD4^+ ^TIL ratios had median survival rates of 74 months versus 25 months, respectively, with a corresponding hazard ratio of 0.30 (95% C.I., 0.16-0.55; *p = *0.0001). These data indicate that CD4^+ ^TILs influence the beneficial effects of CD8^+ ^TIL. The unfavorable effect of CD4^+ ^T cells on prognosis is thought to be due to CD25^+ ^forkhead box P3 (FOXP3)^+ ^regulatory T cells (T_reg_; suppressor T cells), as indicated by the survival of patients with high versus low CD8^+^/T_reg _ratios (58 versus 23 months; hazard ratio = 0.31; 95% C.I., 0.17-0.58; *p *= 0.0002)[4]. This observation strongly suggests that CD4^+^CD25^+^FOXP3^+ ^regulatory T cells within the tumor mass may suppress anti-tumor immunity.

Curiel *et al*. provided the first direct evidence that tumor associated CD4^+^CD25^+^FOXP3^+ ^T_reg _cells correlate to a poor clinical outcome in epithelial ovarian cancer (EOC)[5]. In this study, they revealed a substantial population of CD4^+^CD25^+^CD3^+ ^T cells (10-17% of all T cells) in malignant ascites from 45 untreated EOC patients. CD4^+^CD25^+^CD3^+ ^T cells were concentrated much more in malignant ascites than in the peripheral blood and nonmalignant ascites (0.7-5.0%). Using multicolor confocal microscopy, the study also found a substantial accumulation of CD4^+^CD25^+^CD3^+ ^T cells within the tumor mass among 104 tumor specimens from untreated EOC patients. The percentage of CD4^+^CD25^+^CD3^+ ^T cells was higher in stage II-IV disease than in stage I. In addition, 75% of CD4^+^CD25^+^CD3^+ ^T cells were found in proximity to infiltrating CD8^+ ^T cells, which indicated the possibility of inhibition through physical contact between CD4^+^CD25^+^CD3^+ ^T cells and CD8^+ ^T cells. Furthermore, they confirmed that CD4^+^CD25^+^CD3^+ ^T cells have characteristics of T_reg _cells, which bear the surface phenotype of CD4^+^CD25^+^CD3^+^GITR^+^CTLA4^+^CCR7^+^FOXP3^hi^. These cells also suppressed the proliferation of CD3^+^CD25^- ^T cells, as well as IFN-γ and IL-2 production *in vitro*. Also, they found that T_regs _preferred to accumulate in the tumor mass rather than in tumor-draining lymph nodes. Moreover, the CD4^+^CD25^+ ^T cells in tumor-draining lymph nodes declined from stage I to IV, suggesting they were preferentially recruited to the tumor mass. They also showed that tumor T_regs _were associated with higher risk of death and reduced survival time. In multivariate analysis, individuals with the highest T_reg _content experienced a 25.1-fold risk of death compared to those with the lowest T_reg _content (95% C.I., 6.8-92.1). After controlling for stage of disease and surgical debulking, tumor T_reg _cells were a significant predictor for death and survival in ovarian cancer[5]. Another study showed that high FoxP3 mRNA expression in tumor samples from patients with invasive ovarian cancer had poorer overall survival (27.8 vs. 77.3 months, *p *= 0.0034) and progression-free survival (18 vs. 57.5 months; *p *= 0.0041) when compared with patients with lower FoxP3 mRNA expression.

In Cox multivariate regression analysis, FoxP3 high expression was an independent prognostic factor for both progression-free and overall survival (*p *= 0.004).

These studies strongly suggest that the immune response against ovarian cancer is a significant and independent prognostic factor. It highlights the possibility that favorable anti-ovarian cancer immune response could indeed result in improvement of the clinical outcome[12].

## Ovarian Cancer Immunotherapy as an Effective Treatment Modality: The Hypothesis

Ovarian cancer of epithelial origin is an adenocarcinoma of the epithelial lining of the ovary. Because of the cryptic location of the ovary, ovarian cancer is usually diagnosed after regional or distant metastasis. The major cause of mortality is clinical relapse. Following standard surgery and chemotherapy, immunotherapy may boost the memory anti-tumor immune response to eradicate residual micrometastatic disease and to prevent relapse when given the consolidation therapy. Immunotherapy as a potential approach for treatment of ovarian cancer is based on the following evidence: (1) ovarian cancers express tumor-associated antigens, e.g. HER2/neu[13,14], MUC1[15], OA3[16], membrane folate receptor[17], TAG-72[18], mesothelin[19], NY-ESO-1[20], and sialyl-Tn[21], which can serve as targets for humoral and cellular immune responses; (2) the presence of TILs correlates strongly with survival[2]; (3) ovarian cancers express peptide/MHC complexes, which can be recognized by CD8^+ ^T lymphocytes; (4) and most importantly, the dynamic interaction between host immunity and cancer indicate that the balance between the two forces can be tipped to favor the host immunity, with the ever increasing arsenals of the immunological nature. Taken together, it has been hypothesized that immunotherapy could be an innovative and effective supportive therapy for ovarian cancer.

## Clinical Trials of Immunotherapeutic Strategies Against Ovarian Cancer: the Opportunities

Current immunotherapeutic treatment options for ovarian cancer include but are not limited to therapy with antibodies (Abs) for example against CA125 and idiotypic antibodies, cytokines (such as IFNγ, IL-2), active immunization with gene transduced whole tumor cells, peptide-based vaccines, dendritic cell vaccines and heat shock protein (HSP) vaccines. These modalities are at different phases of clinical investigation and, currently, are not the standard of care. Key clinical studies are summarized in Table 2, some of which we describe in more detail below. Strengths and limitations of approaches are listed in Table 3.

**Table 2:** Findings from Clinical Trials of Immunotherapy for Ovarian Cancer

Strategies	Phase	Immune response	Clinical response	Reference
**Antibody-based vaccine**				
Anti-CA125 (Oregovomab MAb B43.13)	I/II	Increased Ag specific T cells	Improved survival	[25,26,75,76]
Anti-idiotype Ab (ACA-125)	I/II	Induced Ab3, Ab1 and ADCC of CA125^+ ^tumor cells	Improved survival	[28,77]
Anti-HER-2 (trastuzumab, pertuzumab)	I/II	NR*	Stable disease for more than 2.5 months	[78,79]
Anti-MUC-1 idiotypic Ab (HMFG1)	I/II	Induced Humoral Immune Responses	Prolonged survival	[80,81]
**Peptide vaccine**				
HER2/neu	I/II	Developed humoral and T cell immune Response	NR*	[13,14]
NY-ESO-1	I	Induced both humoral and cellular immune responses	NR*	[82,83]
**Cytokine vaccine**				
IL-2	I/II	NR*	Prolonged survival	[84]
IFN-α	I/II	NR*	20% complete and 8% partial response	[85-87]
IFN-γ	I	Increased cytotoxity against tumor associated macrophages	NR*	[32,88,89]
**Tumor cell vaccine**				
Whole tumor cells	I	CD8 T-cell response	No clinical response	[33]
Tumor cells transfected with GM-CSF	I	NR*	Improve survival	[34]
**Dendritic cell vaccine**				
DC pulse with autologous tumor antigen	I	DTH	NR*	[90]
DC pulse with mRNA of FR-α		CD4+ and CD8+ T-cell responses	NR*	[91]
DC/tumour-fusion vaccine	Pre-clinical trial	Elevated serum levels of ANA	NR*	[92]
DC pulse with peptide	Pre-clinical trial	CTL	NR*	[43]
**HSP vaccine**				
Gp96	I	Increased NK cell activity		[unpublished data]

* Not reported

**Table 3:** Summary of the Strengths and Limitations of Ovarian Cancer Immunotherapy

Strategies	Pros	Cons
Antibody-based vaccine	Tumor antigen specific. Easy to produce.	Weak immunogenicity. Not for all individuals.
Peptide vaccine	Safe, stable, and easy to produce and modify.	Poor immunogenicity. HLA restriction.
Cytokine vaccine	Easy to manufacture and administer.	Non-specific immunomodulating only.
Tumor cell vaccine	Convenience, contained tumor antigen pool.	Potential safety concern. Difficult to produce. Difficult to standardize.
Dendritic cell vaccine	Powerful professional antigen presenting cells. May prime both T cells and antibody response.	Difficult to manufacture and standardize.
HSP vaccine	May contain multiple antigens.	Difficult to manufacture and standardize.
Immunomodulation with Treg blockage	Promising strategy	No data on ovarian cancer yet Difficult to completely eliminate Treg.

### Antibody-based vaccines

Antibody-based cancer immunotherapy has now become a standard practice in the treatment of lymphoma and other cancers. CA-125, also known as MUC16 is a well-studied ovarian cancer antigen which was initially identified by Bast, et al. in 1981[22]. CA-125 is a surface glycoprotein antigen, which is elevated in 79% of all patients with ovarian cancer[23] and in 95% of patients with stages III and IV ovarian cancer[24].

Oregovomab (Mab B43.13) is a murine monoclonal antibody that binds to CA-125 with high affinity and can induce both humoral and cellular immune responses against ovarian cancer. Ehlen *et al*. performed a pilot phase II study to examine the immunologic and clinical effect of oregovomab in pretreated patients with recurrent ovarian cancer[25]. More than 50% of patients were successfully induced to generate an anti-CA125 antibody as well as CA125 or oregovomab-specific T cells. Three of thirteen patients had stabilization of disease and survival for more than 2 years. In another phase II trial, the combination of chemotherapy and oregovomab in 20 patients with recurrent epithelial ovarian cancer was studied[26]. Fifteen out of the nineteen patients (79%) developed humoral responses, including human anti-mouse antibodies and antibodies against oregovomab. Two patients (11%) developed anti-CA125 antibodies, whereas 7 of 18 (39%) patients produced CA125 specific T cells. In 5 of 8 (63%) patients, T cell response was specific for autologous tumor, and in 9 of 18 (50%) patients, the T cell response was directed against oregovomab. Patients who had a T-cell immune response showed significantly improved survival.

In addition, many investigators have attempted to use an anti-idiotype antibody to increase immunogenicity. Based on Jerne's network theory, immunization with a given antigen will generate specific antibodies against the antigen (termed Ab1). Ab1 can generate anti-idiotypic antibodies against Ab1, termed Ab2. Some of the anti-idiotypic antibodies (Ab2β) express the internal image of the antigen recognized by the Ab1 antibody and can thus be used as surrogate antigens. Immunization with Ab2β could lead to the development of anti-anti-idiotype antibodies (termed Ab3) that recognize the corresponding original antigen identified by Ab1[27]. Abagovomab (formerly ACA-125) is a mouse anti-idiotype monoclonal antibody whose variable epitope mirrors CA-125. In a phase I/IIb study, 119 patients with advanced ovarian cancer were treated with abagovomab. A specific anti-anti-idiotypic antibody (Ab3) was induced in 81 patients (68.1%). Fifty percent of patients developed a specific anti-CA125 antibody and 26.9% of patients were found to have antibody-dependent cell-mediated cytotoxicity of CA125-positive tumor cells. The median survival rate of all patients was 19.4 months (range: 0.50-56 months). However, Ab3-positive patients showed a significantly longer survival rate (median, 23.4 months; p < 0.0001) compared with Ab3-negative patients (median, 4.9 months)[28]. A second Phase I trial of abagovomab, consisting of 36 patients with recurrent ovarian cancer, compared 9 applications (group L) with 6 applications (group S). Ab3 was induced in all evaluable patients. A more than twofold increase of IFN-γ-expression CA125-specific CD8^+ ^T cells was observed at least once during the immunization in 9 of 12 (75%) patients of group L and 3 of 17 (17.6%) of group S (p = 0,006). However, there was no consistent correlation between the induction of Ab3 and frequencies of CA125-specific CTL and T helper cells[29].

HMFG1 is a murine monoclonal antibody with specificity to MUC1, a cell surface glycoprotein that is expressed by more than 90% of epithelial ovarian cancer and other tumors. In a phase I/II study, 52 patients with epithelial ovarian cancer were treated with yttrium-90-labelled monoclonal antibody HMFG1 administered intraperitoneally. After the completion of conventional surgery and chemotherapy, 21 of the 52 patients had no evidence of residual disease. These data suggest that the survival of patients who received the intraperitoneal antibody was prolonged compared to that of historical controls[30].

### Peptide vaccines

Using peptide as immunogens for immunotherapy has many advantages, since peptides are well defined and the risk for sharing with normal cellular proteins can be minimized. In addition, peptide antigens are easy to manufacture, stable, and can be modified to increase their immunogenicity. However, peptide vaccines usually have poor immunogenicity and need to be administered with adjuvants such as GM-CSF. Disis and her colleagues have performed multiple phase I/II clinical trials using HER2 derived peptides for the treatment of patients with HER2 overexpressing tumors. Consistent HER2-specific T cell response was generated. Moreover, epitope spreading was seen in some patients. The magnitude of the T cell response appears to correlate favorably with the clinical response[13].

NY-ESO-1, another promising cancer-testis antigen, is expressed by more than 40% of advanced epithelial ovarian cancers. Diefenbach *et al*. performed a phase I study to evaluate the effects of vaccination with the HLA-A0201-restricted NY-ESO-1b peptide on patients with high-remission-risk epithelial ovarian cancer, and found that the NY-ESO-1 peptide-based vaccine was safe and induced specific T-cell immunity in both NY-ESO-1 positive and NY-ESO-1 negative patients[31].

### Cytokine vaccines

Exogenously supplied cytokines provide immune regulation and maximize the induction, amplification, and/or effector properties of the desirable immune response in the microenvironment of the vaccination site. Combinations of cytokines and chemotherapeutic agents have been tested against ovarian cancer. For example, Schmeler *et al*. from MD Anderson Cancer Center have recently reported the completion of a phase II study to evaluate the efficacy and toxicity of carboplatin, granulocyte-macrophage colony-stimulating factor (GM-CSF) and recombinant interferon gamma 1b (rIFN-γ 1b) in women with recurrent and platinum-sensitive ovarian, fallopian tube and primary peritoneal cancer[32]. Eligible patients were treated with subcutaneous GM-CSF and rIFN-γ 1b before and after intravenous carboplatin until disease progression or unacceptable toxicity. All patients had measurable disease and a chemotherapy-free interval greater than 6 months. Fifty-nine patients received a median of 6 cycles of therapy (range, 1 to 13 cycles). Median age at enrollment was 61 years (range, 35 to 79 years). Median time to progression prior to enrollment was 11 months (range, 6 to 58 months). Of the 54 patients evaluable for response, 9 (17%) had a complete response, 21 (39%) had a partial response, and 24 (44%) exhibited progressive disease. The overall response rate was 56% (95% CI: 41% to 69%). With a median follow-up of 6.4 months, median time to progression was 6 months. Myeloid derived cells and platelets increased on day 9 of each chemotherapy cycle. The most common adverse effects were bone marrow suppression, carboplatin hypersensitivity, and fatigue. Responders reported improved quality of life. Although it is difficult to evaluate the clinical efficacy in the phase II setting, the safety profile and encouraging response warrant further study of this approach.

### Tumor cell vaccines

In the absence of known tumor antigens, whole tumor cell vaccines offer a simple way to prepare the vaccine which contains a broad tumor antigen repertoire. But whole tumor cells are poorly immunogenic due to their lack of immunostimulatary signals. In order to increase immunogenicity, the whole tumor cell vaccines need to be associated with a specific adjuvant. In a phase I trial, Berd *et al*. modified autologous cancer cells with the hapten, dinitrophenyl (DNP). Administration of the DNP-tumor cell vaccine to patients with metastatic melanoma induced inflammation in metastatic sites. Histologically, most of the infiltration of T lymphocytes were CD8^+ ^cells[33]. Investigators have tried to modify tumor cell vaccines by transducing GM-CSF into tumor cells. Nemunaitis *et al*. conducted a phase I/II multicenter trial in patients with early and advanced stage non-small-cell lung cancer. Vaccines were successfully manufactured for 67 patients, and 43 were vaccinated. Survival in patients receiving vaccines secreting higher amounts of GM-CSF (median survival = 17 months, 95% CI; 6 to 23 months) was significantly longer than in patients receiving vaccines secreting less GM-CSF (median survival = 7 months, 95% CI; 4 to 10 months) (p = 0.028)[34].

### Dendritic cell vaccines

Dendritic cells (DCs) are major professional antigen-presenting cells which control primary and secondary immune responses to various exogenous antigens through antigen cross-presentation and cross-priming of T cells[35,36]. DCs also play important roles in establishing anti-tumor immunity and autoimmunity [37-39], both of which are immune responses to self-antigens through the breakdown of immune tolerance. Because DCs have a potential to induce antigen-specific anti-tumor immunity, several clinical trials of cancer immunotherapy using DC vaccines have been performed[40,41]. Gong *et al*. used a tumor cell/DC fusion strategy[42]. In this study, human ovarian cancer cells were fused to human DCs, and they found that the fused cells were functional in stimulating the proliferation of autologous T cells, inducing cytolytic T cell activity and the lysis of autologous tumor cells by a MHC class I-restricted mechanism[42]. Brossart *et al*. treated patients with advanced breast and ovarian cancer with autologous DCs pulsed with HER-2/neu- or MUC1-derived peptides. In 5 of 10 (50%) patients, peptide-specific cytotoxic T lymphocytes (CTLs) were generated after vaccination. The major CTL response *in vivo *was induced with the HER-2/neu-derived E75 and MUC1-derived M1.2 peptide. The DC vaccinations were well tolerated with minimal side effects[43].

### Heat shock protein vaccines

HSPs are best known as molecular chaperones, which play vital roles in assisting protein folding[44]. A number of mammalian HSPs (gp96, HSP90, HSP70, calreticulin, HSP110, grp170), when isolated from tumor cells, have been shown to elicit tumor-specific immunity, and when isolated from virus-infected cells, have been demonstrated to elicit virus-specific immunity[45,46]. The immunity in each case is specific to the individual tumor (or virus-infected cell) that was used as the source of the HSP preparation. A large number of clinical trials have been carried out to determine if tumor-derived HSP preparations are able to elicit tumor-specific immunities. Results from human clinical trials in our institution and others in melanoma, renal cell cancer, chronic myelogenous leukemia and other diseases are consistent with the murine experience [47-50].

The effects of HSPs against a wide spectrum of cancers, across species, appear to be related to three key features: (1) HSPs that are isolated from cancer cells, although pure and homogenous, are bound to a wide array of peptides, including antigenic tumor-specific peptides. Therefore, pure HSPs isolated from a tumor cell also contain the entire antigenic peptides from this cell[46]. (2) HSP-peptide complexes can interact with a conserved receptor molecule CD91 on the surface of DCs[51]. These complexes are internalized by DCs, and the peptides that were chaperoned by HSPs are cross-presented by MHC I molecules of the DCs. These MHC I-peptide complexes now stimulate naïve CD8^+ ^T cells that mediate the anti-tumor activity. (3) HSP-DC interaction also leads to the activation of DCs, resulting in the production of proinflammatory cytokines and upregulation of co-stimulatory molecules which are necessary for the activation of T cell responses[46].

Our laboratory conducted a pilot study on the roles of the autologous ovarian cancer-derived gp96-peptide complex in the treatment of patients with stage III and IV ovarian cancer in the consolidation setting[52]. We hypothesized that effective immune intervention at the time of minimal residual disease is the ideal means to prevent relapses of this disease. Patients who completed the standard therapy with no disease progression were eligible to receive the vaccine. Seven patients (6 with stage IIIc disease, 1 with stage IIIb cancer) completed the gp96 injection at 25 μg i.d., weekly for 8 weeks. Grade II or higher toxicity was not observed. No clinical evidence of autoimmunity was found. Five out of seven patients showed increased frequency of IFNγ-producing cells in the peripheral blood against gp96-pulsed autologous antigen-presenting cells (APCs) that are MHC class I-dependent. Of interest, 6 out of 7 patients demonstrated increased NK cell activity, measured by IFNγ ELISPOT against NK cell target K562 cells. This finding is consistent with our prior study that demonstrated a significant increase of NK cell activity in patients with chronic myeloid leukemia (CML) after vaccination with HSP70, which led us to hypothesize that HSPs are able to mediate NK-DC cross-talk[49,53]. Our results demonstrated that a HSP-based vaccine is feasible, well tolerated and is able to induce favorable immune responses against ovarian cancer.

## What are the Challenges for Ovarian Cancer Immunotherapy?

Although various immunotherapeutic approaches have been examined for the treatment of ovarian cancer, it remains true that no such therapy has entered into the clinical standard of care. Below we outline several challenges that need to be overcome.

When patients are diagnosed with cancer, by definition, the tumor has "escaped" the immune system, having passed the phases of "elimination" and "equilibrium". Although there is no shortage of ovarian cancer antigens due to genomic instability and accumulation of mutated genes at this point, the generation of immune response against these antigens is likely unproductive in the late stage, due to multiple immune tolerance mechanisms such as Treg infiltration in the tumor bed, general immune suppression from immunosuppressive cytokines by tumor cells, and down-regulation of MHC class I molecules on the tumor cells. Also, myeloid-derived suppressor cells (MDSC) and tumor-associated macrophages (TAM) create an immunosuppressive environment that leads to suppress T cell responses [54-56]. Thus, multiple immunological "brakes" need to be lifted to augment productive immune response. Currently, clinical studies examine one parameter at a time, which is perhaps too little too late. Combined immunotherapeutic modalities need to be seriously considered in order to break the "glass is half empty" reality of the current immunotherapy landscape in the treatment of ovarian cancer.

There are also practical challenges. It is an unclear and certainly not a trivial question to ask how immunotherapy shall be incorporated into conventional therapy. Surgery and chemotherapy are all seriously immunosuppressive at certain circumstances [57,58], making them very difficult to combine with immunotherapy. Hence, the field is moving toward immunological intervention of patients after the completion of conventional therapy. One bold question is whether or not immunotherapy shall be moved up front, to be followed by surgery and chemotherapy. This seemingly counter-intuitive idea is founded on the premise that antigen-specific memory cells might well withstand conventional chemotherapy. Better yet, cancer vaccines should ideally be given to women in the high-risk category who have not yet been diagnosed with clinical cancer, during the "equilibrium" phase. This last scenario also depends, in part, on the ability of the medical field to screen and diagnose ovarian cancer much earlier than we are currently able to achieve. Lastly, it is worthwhile to reiterate that combined immunological modalities may be the best way to move forward. This approach demands the collaboration of investigators and the creativity of regulatory agencies such as the FDA for approval of novel combinations of various approaches in situations where none of these approaches alone has been shown to be effective yet.

## Conclusion and Perspectives

In light of highly promising advancements in the science of immunotherapy against ovarian cancer coupled with encouraging results from numerous clinical trials, we suggest that bold steps need to be taken to further this area of research. First, a more permissive regulatory climate is needed to allow investigators to combine various non-proven modalities in hopes of finding an effective combination. Second, we should focus on finding biomarkers for early diagnosis or prognosis and individual treatment. Serum proteomics applications could identify blood-based biomarkers for early diagnosis and prognosis[59], and tissue proteomics could help to define targets for individualized treatment. Third, we should debate the merits to move immune intervention ahead of conventional therapy or even to high-risk patients in the prophylactic setting. Finally, resources and funding must be given to support the important translational groundwork by cancer immunologists and physician scientists. Without these critical steps, we might face the same uncertainty about therapy against this dreadful disease for years to come.

## Competing interests

The authors declare that they have no competing interests.

## Authors' contributions

BL participated in literature review and wrote the manuscript. BL, HS, RS, ZL conceived the concept. JN, CR, ZL, BL contributed the phase I trial data for heat shock protein vaccine. All authors participated in revising the manuscript and approved the final manuscript.
